# Recurrent ventilator-associated pneumonia in severe Covid-19 ARDS patients requiring ECMO support

**DOI:** 10.1186/s13613-024-01295-1

**Published:** 2024-04-25

**Authors:** Elena Collado-Lledó, Quentin Moyon, Juliette Chommeloux, Marc Pineton de Chambrun, Guillaume Hékimian, Ouriel Saura, David Lévy, Matthieu Schmidt, Alain Combes, Charles-Edouard Luyt, Lucie Le Fevre

**Affiliations:** 1 Sorbonne Université, Assistance Publique–Hôpitaux de Paris, Hôpital Pitié–Salpêtrière, Médecine Intensive Réanimation, Assistance Publique, Hôpitaux de Paris (AP-HP), Paris, France; 2 INSERM, UMRS_1166-iCAN, Institute of Cardiometabolism and Nutrition, Paris, France

**Keywords:** Ventilator-associated pneumonia, ECMO, COVID-19

## Abstract

**Objective:**

To describe ventilator-associated pneumonia (VAP) recurrence in COVID-19 patients requiring extracorporeal membrane oxygenation (ECMO) support, and to evaluate the impact of antimicrobial treatment duration of the first VAP episode on VAP recurrence.

**Methods:**

Adult patients with COVID-19 severe pneumonia on ECMO admitted between March 2020 and January 2022 were retrospectively included. Primary outcome was incidence of VAP recurrence, and secondary outcome was the impact of duration of antimicrobial treatment on VAP recurrence.

**Results:**

Among the 252 included patients, 226 (90%) developed a first VAP. Sixteen had lung abscess and were excluded, leaving 210 patients. VAP recurrence occurred in 172 patients (82%), with a median (IQR) time from first VAP to recurrence of 10 (7–13) days. *Pseudomonas aeruginosa* and Enterobacteriaceae were respectively responsible for 28% and 52% of first VAP, and 51% and 62% of first recurrence episodes. Among the 210 patients with a first VAP, 158 (75%) received a short course of antibiotics [< 8 days, median (IQR) duration 6 (5–7) days] and 52 (25%) received a prolonged course of antibiotics [≥ 8 days, median (IQR) duration 9 (8–10) days]. Estimated cumulative incidence of VAP recurrence, taking into account death and extubation as competing risks, was not different in patients with short– and prolonged–antimicrobial treatment.

**Conclusions:**

In patients with severe Covid-19–ARDS requiring ECMO support, VAP recurrence occurs frequently, with Enterobacteriaceae and *Pseudomonas aeruginosa* as predominant causative microorganisms. An antimicrobial treatment of ≥ 8 days for the treatment of first VAP episode did not reduce the risk of VAP recurrence, as compared to shorter duration.

**Supplementary Information:**

The online version contains supplementary material available at 10.1186/s13613-024-01295-1.

## Background

Acute respiratory distress syndrome (ARDS) secondary to severe coronavirus infection disease 2019 (Covid-19)-associated pneumonia has become a major cause of admission in intensive care units (ICU) since the outbreak of the worldwide pandemic [1]. The most severe forms can rapidly evolve to profound hypoxemia despite lung-protective mechanical ventilation, needing extracorporeal membrane oxygenation (ECMO) support [2, 3]. These critically ill patients, who need prolonged mechanical ventilation (MV), prone-positioning, sedation and neuromuscular blockade for weeks, are at high risk of developing ventilator-associated pneumonia (VAP) [1, 4]. Previous reports indicated that Covid-19 patients had a higher prevalence of VAP compared with ARDS of other causes, suggesting that Covid-19 particular pathophysiology may also play a key role in VAP development [5–9], and that the most severe patients, those on ECMO, may develop VAP more frequently [5]. Microbiology of VAP has also been described, with most studies indicating that Enterobacteriaceae and *Pseudomonas aeruginosa* are the main pathogens responsible for the first VAP episode in Covid-19 patients [5–7, 9, 10]. However, little is known about the characteristics of VAP recurrences in patients with Covid-19, especially in the setting of severe ARDS requiring ECMO support. We hypothesized that these patients may have a high risk of developing more than one episode of VAP, and that the bacterial species and drug resistance profiles of these following VAP episodes may be different from the first one.

We therefore conducted a retrospective study to evaluate the characteristics of VAP recurrences and outcomes in all patients admitted to our ICU (a tertiary referral center for ECMO) for virologically confirmed Covid-19–ARDS requiring ECMO since the beginning of the first wave in France (March 2020) and all along the different pandemic waves until February 2022.

## Materials and methods

### Patients

All consecutive adult patients with RT-PCR confirmed severe SARS-CoV-2 ARDS [11] requiring veno-venous ECMO admitted to our ICU between March 2020 and January 2022 were included. Patients with other types of ECMO support (veno-arterial-venous, veno-arterial) were also included as long as they presented with SARS-CoV-2 ARDS, whereas patients who received only veno-arterial ECMO support for cardiogenic shock were excluded. Since our goal was to describe risk factors for VAP recurrence, patients who developed lung abscess at the time of first VAP episode were excluded. Patients that were described in a previously published study were included in the present study [5].

### VAP diagnosis and treatment

All ventilated Covid-19 patients suspected of developing VAP immediately underwent fiberoptic bronchoscopy, using bronchoalveolar lavage (BAL) to collect distal respiratory samples from the area in which purulent secretions were most abundant. VAP was diagnosed in patients under MV for at least 48 h who were clinically suspected of having developed VAP, and had significant quantitative growth (≥ 10^4^ colony-forming units (CFU)/mL) of at least one pathogen on BAL fluid sample [12–14]. As it may be difficult to suspect VAP in patients with ARDS under ECMO support, a high vigilance towards suspicion of VAP was maintained throughout the study period and bronchoscopic samples were obtained as soon as a patient became febrile, had purulent tracheal secretions, deteriorated clinically (needing introduction of vaso-active drugs or increasing their dose), or showed increasing white blood cells count, even when progression of lung infiltrates was uncertain. Extreme vigilance for VAP recurrence was maintained, and fiberoptic bronchoscopy with BAL was again performed as soon as any intercurrent event imposed a change of antimicrobial regimen. Empirical antimicrobial treatment was started according to the recent French guidelines. Therapeutic drug monitoring was part of routine care, to the clinician’s discretion. Treatment duration was left to the clinician’s discretion.

### Definitions

#### VAP recurrence

Patients were considered to have a VAP recurrence when a new VAP diagnosis was made according to the above-described criteria after a period of partial or complete resolution of the previous VAP episode symptoms. VAP recurrences were classified as relapse, persistent infection or superinfection. The definitions used for recurrences were those used by Chastre et al. in the Pneuma trial [15]. A VAP recurrence was considered a **relapse** when 1) at least one of the causative microorganisms (same genus and species) of the previous VAP grew over 10^4^ CFU/mL from the BAL fluid, and 2) antimicrobial regimen administered for the previous VAP episode ended more than 48 h before onset of the current recurrence episode. A VAP recurrence was classified as **persistent infection** when 1) at least one of the causative microorganisms of the previous VAP grew over 10^4^ CFU/mL from the BAL fluid, and 2) antimicrobial regimen administered for previous VAP episode was still ongoing or ended less than 48 h before onset of the current recurrence episode. When durably positive bacterial growth of BAL fluid with the same microorganism was attributed to lung abscess, patients were not considered as having a VAP recurrence, but as one long-lasting unique episode of necrotizing VAP. Lastly, VAP recurrence was considered a **superinfection** when due to a new microorganism, i.e., none of the causative microorganisms of the previous VAP grew significantly from the BALF, whatever the time between the 2 VAP episodes.

#### Antibiotic resistance profiles

Enterobacteriaceae were classified into susceptible, extended-spectrum beta-lactamase (ESBL)-producing, AmpC cephalosporinase hyperproducing, and carbapenemase-producing strains. *Staphylococcus* (*S. aureus* and coagulase-negative) were classified into methicillin-susceptible and methicillin-resistant strains. *Pseudomonas aeruginosa* strains were classified as difficult-to-treat when they exhibited non-susceptibility to all of the following: piperacillin-tazobactam, ceftazidime, cefepime, aztreonam, meropenem, imipenem-cilastatin, ciprofloxacin, and levofloxacin [16].

#### Multidrug-resistant organism acquisition

Multidrug-resistant organisms (MDRO) carriage screening consisted of testing for the presence of ESBL-producing Enterobacteriaceae and carbapenemase-producing Gram-negative bacilli from rectal swabs by PCR and bacterial growth. Patients were screened for MDRO rectal carriage at admission and weekly afterwards for the entire ICU stay. Patients with negative MDRO screening on admission and either rectal screening or clinical sample positive for MDRO during ICU stay were considered as having MDRO acquisition.

### Data collection and analysis

Data prospectively recorded in each patient’s medical chart included demographic characteristics, SARS-CoV-2 vaccination status, comorbidities and severity scores (Simplified Acute Physiology Score (SAPS) II and Sequential Organ-Failure Assessment (SOFA) score) at ICU admission. SARS-CoV-2 variant was recorded or inferred from epidemiological data as described above. Date of symptoms onset, hospital and ICU admission, MV onset and start of ECMO support were also obtained, as well as the use of immunomodulatory drugs (steroids, tocilizumab) before the first VAP. For each VAP episode, we recorded the date and type of respiratory sample, quantitative bacterial growth and antibiotic resistance profile of each pathogen. Antimicrobial regimen duration with the start and end dates of each antimicrobial drug received were also obtained, including both empirical and definitive treatments. The identification of an abscess on CT scan was also noted for each VAP episode. Additionally, antimicrobial regimens administered for bloodstream infections and ECMO cannula-related infections were also collected, in order to measure antibiotic exposure. Data regarding antimicrobial blood measurement, when available, were retrieved. Dosage was considered adequate when this latter was above the European committee on antimicrobial susceptibility testing (EUCAST) breakpoints for the pathogen responsible for VAP. Finally, data concerning the duration of ECMO support, MV and ICU stay, as well as ICU mortality, were also recorded.

### Outcomes

The primary outcome was the incidence of VAP recurrences, their microbiological description as compared to first VAP and their distribution between relapses, persistent infections and superinfections. Secondary outcomes included the impact of antimicrobial treatment duration of first VAP on the incidence of a recurrence and on antibiotic consumption. For this, patients were grouped according to the duration of antimicrobial treatment of the first VAP episode: patients having received up to 7 days of antimicrobial treatment (hereafter called “short” duration) and patients having received 8 days or more of antimicrobial treatment (hereafter called “prolonged” duration).

### Statistical analyses

Patient characteristics are expressed as n (%) for categorical variables or median (interquartile range, IQR) for continuous parameters. Between-group comparisons were analyzed using Student’s *t* test or Mann–Whitney *U* tests according to variable’s distribution, i.e., normal or not, respectively, for continuous variables. Between-group differences were assessed with the chi-square test or Fisher’s exact test for nominal variables.

Incidence of VAP recurrence in the two groups of patients (“short” or “prolonged” treatment of the first VAP, as described above) was compared using an estimated cumulative incidence function to take into account competing factors (death or extubation) as previously described [17, 18]: cumulative incidence of VAP recurrence, extubation and death were estimated in each group, taking into account only the first event, and compared. Day 0 was defined as occurrence of first VAP episode. To further explore factors associated with recurrence, a propensity score was constructed using multivariable analysis, with VAP recurrence as the dependent variable. Variables included in the multivariable model were those with a P-value < 0.2 in univariable analysis, namely delta variant as the SARS-CoV-2 serotype responsible for infection, tocilizumab use, need for renal replacement therapy and polymicrobial VAP. The propensity score for developing VAP recurrence, calculated for each patient based on this multivariable analysis, was then included in the multivariable model of factors associated with VAP recurrence (Fine and Gray model).

Antibiotic-free days were defined as the number of days alive without any antibiotic 60 days after the first VAP episode. Patients dying before day 60 were assigned zero antibiotic-free days. Data regarding antibiotic exposure were not available for 82/226 patients (36%); 67 patients in the short-duration group and 15 patients in the prolonged-duration group. For these 82 patients (all alive at day 60), missing data corresponded to days spent outside the hospital, after hospital discharge and before day 60, and were arbitrarily imputed as days without antibiotic. All analyses were computed at a two-sided α level of 5% using SPSS Version 23 (IBM SPSS, Chicago, IL) and R software, version 4.2.2 (R Foundation).

### Ethics

In accordance with the current French law, informed written consent for data collection and analyses was not obtained because this observational study did not modify patient management. Patients or relatives were informed about anonymous data collection and their possibility to decline inclusion. The database is registered by the Commission Nationale de l’Informatique et des Libertés (CNIL) under registration number 1950673.

## Results

**Fig. 1:**
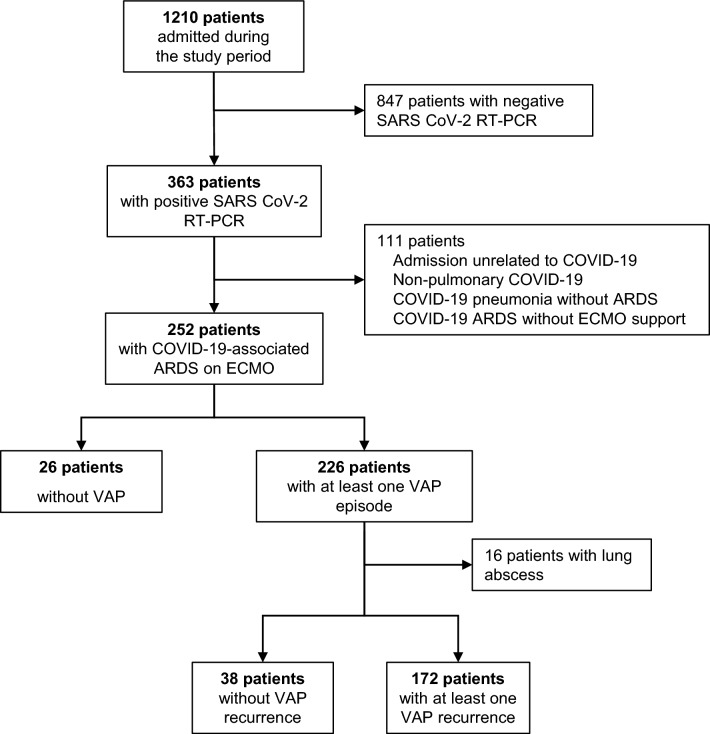
Study flowchart. *SARS CoV-2* severe acute respiratory syndrome coronavirus-2, *RT-PCR* reverse transcriptase polymerase chain reaction, *ARDS* acute respiratory distress syndrome, *ECMO* extracorporeal membrane oxygenation, *VAP* ventilator-associated pneumonia

During the study period, 252 patients with severe Covid-19 ARDS requiring ECMO support were admitted to our ICU (Fig. 1). Patients’ characteristics at ICU admission are reported in Table 1. They were mostly men, and a vast majority of patients received VV-ECMO support. Most patients admitted after April 2020 received early dexamethasone regimen in accordance with evolving knowledge throughout the pandemic. VAP incidence was high with 226 patients (90%) developing at least one VAP, after a median (IQR) time of 7 (3–11) days after intubation. Among these 226 patients, 16 had a lung abscess and were excluded from the analysis, leaving 210 patients with at least one VAP episode. Details on antimicrobial treatments received are given in the Additional file 1: eTable 1. The rates of appropriate empiric treatment and its nature (agent received, combination or not), as well as definitive treatment were similar in patients with or without recurrence. Moreover, among the patients who had antibiotic serum level measurement, the proportion of patients with adequate serum level was similar in patients with and without recurrence (Additional file 1: eTable 2).

**Table 1:** Patients’ characteristics, procedures during ICU stay and outcomes

Parameter	Patients N = 252
Age, years	50 (42–57)
Male sex	173 (69)
Body mass index, kg/m^2^	31.9 (27.8–38.1)
Comorbidities	
Hypertension	97 (39)
Diabetes	77 (31)
Immunocompromised	18 (7)
Vaccinated against SARS CoV-2	9 (4)
Admission SAPS II	61 (52–68)
Admission SOFA score	12 (9–13)
Time from first symptoms to ICU admission, days	7 (5–10)
Time from ICU admission to intubation, days	2 (0–6)
Time from intubation to ECMO, days	4 (1–7)
Type of ECMO support	
VV	247 (98)
Femoro-jugular	238 (96)
Femoro-femoral	9 (4)
VA	3 (1)
VAV	2 (1)
High-flow nasal oxygen before intubation	175 (69)
Non-invasive ventilation before intubation	103 (41)
Prone-positioning before ECMO	229 (91)
Prone-positioning on ECMO	193 (77)
Immunomodulatory drugs	
Tocilizumab	25 (10)
Dexamethasone	188 (75)
Number of VAP episodes	
0	26 (10)
1	44 (18)
2	49 (19)
3	46 (18)
4	34 (14)
≥ 5	53 (21)
Time from ICU admission to first VAP, days	10 (6–15)
Time from intubation to first VAP, days	7 (3–11)
Duration of ECMO support, days	28 (12–46)
Duration of mechanical ventilation, days	44 (25–63)
ICU length of stay, days	49 (31–71)
Successful ECMO weaning	151 (60)
ICU mortality rate	111 (44)

Results are expressed as median (IQR) or n (%)

*SARS CoV-2* severe acute respiratory syndrome coronavirus-2, *SAPS II* simplified acute physiology score II, *SOFA* sepsis-related organ failure assessment, *ECMO* extracorporeal membrane oxygenation, *VV* veno-venous, *VA* veno-arterial, *VAV* veno-arterial-venous, *VAP* ventilator-associated pneumonia, *ICU* intensive care unit

There was no difference in VAP incidence when taking into account the Covid-19 wave or the SARS-CoV-2 variant (data no shown).

Data on VAP episodes are reported in Table 2. Among the 210 first VAP episodes, 31% were polymicrobial, and Enterobacteriaceae were the most frequent pathogens retrieved. VAP recurrence occurred in 172 patients out of the 210 patients (82%) who had a first VAP. Among the 172 patients with at least one recurrence, 155 (90%) occurred while the patient was on ECMO support and 17 (10%) occurred after ECMO withdrawal. Median (IQR) time between first VAP onset and first recurrence was 10 days (IQR 7–13). Among the 172 first recurrences, 108 (63%) involved at least one of the pathogens identified on first VAP: 55 (32%) were persistent infections and 53 (31%) were relapses.

**Table 2:** Characteristics of ventilator-associated pneumonia episodes

Characteristics	1st VAP (n = 210)	1st recurrence (n = 172)	All following VAP (n = 292)
Time since previous VAP, days	–	10 (7–13)	11 (8–15)
Polymicrobial VAP	65 (31)	64 (37)	113 (39)
Pathogens*			
* Enterobacteriaceae*	109 (52)	106 (62)	157 (54)
Inducible AmpC *Enterobacteriaceae*	41 (38)	43 (41)	70 (45)
ESBL-producing *Enterobacteriaceae*	20 (18)	31 (29)	54 (34)
Carbapenemase-producing *Enterobacteriaceae*	3 (3)	7 (7)	10 (6)
Non-fermenting GNB	75 (36)	112 (65)	208 (71)
* Pseudomonas aeruginosa*	59 (79)	87 (78)	176 (85)
Difficult-to-treat strain^†^	1 (2)	7 (8)	39 (22)
* Stenotrophomonas maltophilia*	12 (16)	16 (14)	26 (13)
* Acinetobacter spp.*	3 (4)	8 (7)	5 (2)
Gram-positive cocci	67 (32)	21 (12)	38 (13)
* Staphylococcus aureus*	39 (58)	12 (57)	20 (53)
Methicillin susceptible	33 (85)	8 (67)	14 (70)
Methicillin resistant	6 (15)	4 (33)	6 (30)
* Enterococcus spp.*	11 (16)	7 (33)	17 (45)
* Streptococcus spp.*	12 (18)	1 (5)	1 (3)
Abscess	–	13 (8)	14 (5)
Type of recurrence			
Persistent infection	–	55 (32)	142 (49)
Relapse	–	53 (31)	102 (35)
Superinfection	–	64 (37)	48 (16)

Results are expressed as median (IQR) or n (%)

*VAP* ventilator-associated pneumonia, *ESBL* extended-spectrum beta-lactamase, *GNB* Gram-negative bacilli

*Total number of pathogens exceeds the number of VAP due to polymicrobial cases, where more than one pathogen grew at a concentration > 10^4^ CFU/mL

^†^Strains were considered difficult-to-treat when they exhibited non-susceptibility to all of the following: piperacillin-tazobactam, ceftazidime, cefepime, aztreonam, meropenem, imipenem-cilastatin, ciprofloxacin, and levofloxacin

The burden of *P. aeruginosa* increased noticeably from first VAP (29%) to first recurrence (51%), accompanied by a surge in its resistance rate, since difficult-to-treat strains increased from 1 to 8%. The proportion of Enterobacteriaceae increased moderately (from 50% of first VAP to 60% of first recurrence) with ESBL-production rate rising from 18 to 29% (Table 2).

**Fig. 2:**
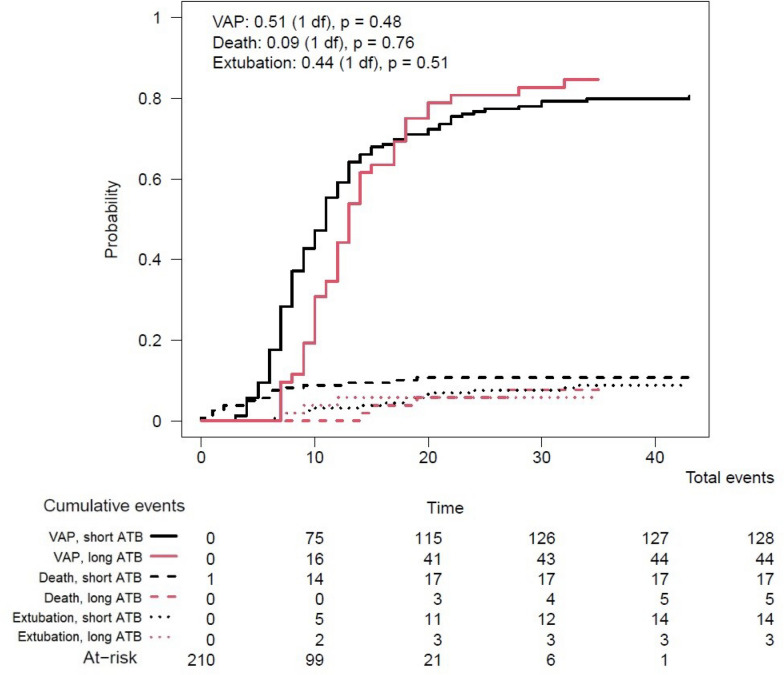
Estimated cumulative incidence of VAP recurrence, extubation or death in patients with short (< 8 days, short ATB) or long (≥ 8 days, long ATB) duration of antimicrobial treatment of first VAP episode. P values are given for comparisons between short and long duration of antimicrobial treatment groups. *VAP* ventilator-associated pneumonia recurrence, *ATB* antimicrobial treatment

Among the 210 patients presenting with a first VAP, 158 (74%) received a short-course of antimicrobial treatment [median (IQR) duration 6 (5–7) days] and 52 (26%) received a prolonged-course of antimicrobial treatment [median (IQR) duration 9 (8–10) days]. Characteristics of patients according to duration of treatment of first VAP episode are presented in Table 3. In a survival model comparing the short– and prolonged– duration groups, the estimated cumulative incidence of developing a VAP recurrence, taking into account death and extubation as competing risks, was not significantly different (P = 0.42) (Fig. 2). The median (IQR) number of antibiotic-free days at day 60 was 29 (0–41) in the short-duration group and 15 (0–37) in the prolonged-duration group (P = 0.1). Multivariable analysis of factors associated with VAP recurrence (Fine and Gray model) displayed similar results (Table 4): factors associated with VAP recurrence were prone positioning during ECMO and *Pseudomonas aeruginosa* as the pathogen responsible for VAP. A duration of antimicrobial treatment of first episode < 8 days was not associated with recurrence, even when forced into the multivariable model (HR 0.25, 95% CI 0.04–1.72).

**Table 3:** Characteristics of patients according to the duration of antimicrobial treatment of the first episode

Parameter	Overall population N = 210	Duration of treatment < 8 days N = 158	Duration of treatment ≥ 8 days N = 52	P value
Age, years	51 (43–58)	51 (43–58)	51 (38–58)	0.2
Male sex	145 (69)	108 (68)	37 (71)	0.7
Body mass index, kg/m^2^	32 (28–38)	32 (28–38)	32,5 (28–43)	0.9
Comorbidities				
Hypertension	84 (40)	66 (42)	18 (35)	0.4
Diabetes	65 (31)	48 (30)	17 (33)	0.8
Immunocompromised	15 (7)	9 (6)	6 (12)	0.2
Vaccinated against SARS CoV-2	5 (2)	3 (2)	2 (4)	0.6
Admission SAPS II	61 (52–68)	61 (53–67)	62 (50–71)	0.8
Admission SOFA score	12 (9–13)	12 (9–13)	12 (8–13)	0.7
Time from first symptoms to ICU admission, days	7 (5–10)	8 (5–10)	7 (4–10)	0.4
Time from ICU admission to intubation, days	3 (0–6)	2 (0–6)	4 (1–7)	0.07
Time from intubation to ECMO, days	4 (2–7)	4 (2–7)	4 (2–7)	0.9
Type of ECMO support				
VV	207 (99)	155 (98)	52 (100)	1.0
Femoro-jugular	202 (98)	153 (99)	49 (94)	0.1
Femoro-femoral	5 (2)	2 (1)	3 (6)	0.1
VA	2 (1)	2 (1)	0 (0)	1.0
VAV	1 (0)	1 (1)	0 (0)	1.0
High-flow nasal oxygen before intubation	146 (70)	106 (67)	40 (77)	0.2
Non-invasive ventilation before intubation	82 (39)	58 (37)	24 (46)	0.2
Prone-positioning before ECMO	198 (94)	149 (94)	49 (94)	1.0
Prone-positioning on ECMO	169 (81)	130 (82)	39 (75)	0.3
Immunomodulatory drugs				
Tocilizumab	19 (9)	11 (7)	8 (15)	0.09
Dexamethasone	158 (75)	116 (73)	42 (81)	0.3
Number of VAP episodes				
1	38 (18)	30 (19)	8 (15)	0.6
2	48 (23)	30 (19)	18 (35)	0.02
3	42 (20)	31 (20)	11 (21)	0.8
4	32 (15)	26 (17)	6 (12)	0.4
≥ 5	50 (24)	41 (26)	9 (17)	0.2
Type of recurrence				
Persistent infection	55 (26)	35 (22)	20 (39)	0.02
Relapse	53 (25)	39 (25)	14 (27)	0.7
Superinfection	64 (31)	54 (34)	10 (19)	0.04
Duration of mechanical ventilation, days	47 (30–66)	48 (29–67)	47 (30–62)	0.8
Ventilator-free days at day 60*, days	0 (0–20)	0 (0–21)	0 (0–18)	0.6
ICU length of stay, days	53 (36–72)	53 (37–72)	52 (35–68)	0.8
Successful ECMO weaning	133 (63)	103 (65)	30 (58)	0.3
ICU mortality rate	86 (41)	61 (39)	25 (48)	0.2

Results are expressed as median (IQR) or n (%)

*SARS CoV-2* severe acute respiratory syndrome coronavirus-2, *SAPS II* simplified acute physiology score II, *SOFA* sepsis-related organ failure assessment, *ECMO* extracorporeal membrane oxygenation, *VV* veno-venous, *VA* veno-arterial, *VAV* veno-arterial-venous, *VAP* ventilator-associated pneumonia, *ICU* intensive care unit

*Calculated 60 days after first VAP episode onset

**Table 4:** Uni- and multivariable analyses of factor associated with VAP recurrence, taking into account death and extubation as competing events (Fine and Gray competitive risk analysis)

Variables	Univariable analysis		Multivariable analysis	
	HR (95% CI)	P value	HR (95% CI)	P value
Age > 51 years	0.94 (0.71–1.25)	0.7		
Female sex	1.13 (0.83–1.53)	0.5		
Body mass index > 32 kg/m^2^	0.91 (0.68–1.21	0.5		
Immunocompromised	1.10 (0.57–2.12)	0.8		
Diabete	1.12 (0.82–1.53)	0.5		
Chronic lung disease	0.92 (0.62–1.36)	0.7		
Admission SAPS II score > 61	1.04 (0.78–1.38)	0.8		
Delta variant responsible for Covid-19	1.25 (0.92–1.71)	0.2		
Dexamethasone use	1.00 (0.70–1.41)	0.9		
Tocilizumab use	1.86 (1.12–3.08)	0.02	1.45 (0.76–2.77)	0.3
Glucorticoids use > 40 mg/day	1.16 (0.73–1.84)	0.5		
Prone positionning during_ECMO	1.75 (1.17–2.62)	0.006	1.74 (1.17–2.60)	0.006
Need for renal replacement therapy	1.05 (0.79–1.40)	0.7		
Pseudomonas aeruginosa responsible for VAP	1.74 (1.25–2.42)	0.001	1.74 (1.17–2.60)	0.006
Polymicrobial_VAP	1.04 (0.77–1.40)	0.8		
Adequate initial antimicrobial treatment	1.06 (0.62–1.80)	0.8		
Empirical treatment including an aminoglycosides	0.85 (0.62–1.18)	0.3		
Empirical treatment including an anti-MRSA agent	0.85 (0.51–1.40)	0.5		
Combination therapy for definitive treatment	1.21 (0.63–2.31)	0.6		
Treatment of first episode < 8 days*	1.07 (0.81–1.42)	0.6	0.25 (0.04–1.72)	0.2
Propensity score on factors associated with treatment < 8 days	0.21 (0.05–0.86)	0.03	1.22 (0.91–1.63)	0.2

*SAPS II* simplified acute physiology score II, *ECMO* extracorporeal membrane oxygenation, *VV* veno-venous, *VA* veno-arterial, *VAV* veno-arterial-venous, *VAP* ventilator-associated pneumonia, *MRSA* methicillin-resistant *Staphylococcus aureus*

Including the 16 patients with lung abscess display similar results (data not shown). Weekly rectal screening for MDRO was available for 198 patients of our cohort (85%). Thirty-seven patients (16%) acquired rectal colonization with ESBL-producing Enterobacteriaceae during their stay, among which 6 patients also acquired carbapenemase-producing Enterobacteriaceae. Sixteen (43%) among the 37 patients who acquired MDRO developed subsequent MDRO VAP.

## Discussion

In this cohort of 252 patients with severe Covid-19 ARDS under ECMO support, a vast majority (90%) of patients developed a first VAP episode during their ICU stay, and most of them (81%) had at least one VAP recurrence. Persistent infections and relapses represented together 63% of first recurrences and 84% of the following ones, outlining the difficulty to durably eradicate pathogens from the lung in this population. Prolonging the duration of antimicrobial treatment for more than 7 days did not seem to prevent the risk of VAP recurrence in our study.

Previously published cohorts of Covid-19 ARDS patients also described VAP incidences that were significantly higher compared to other causes of ARDS, especially when compared to influenza ARDS [5–7] or to non-viral pneumonia requiring MV [6, 8, 9]. VAP incidence in our cohort matches the incidence reported in previously published cohort [5], suggesting that the remarkably high incidence of VAP in the first wave cohort is not only a consequence of altered compliance with the ventilation care bundle in a situation of staff and equipment shortages. Our microbiological data is consistent with published work on Covid-19 ARDS from other centers [5, 9, 10, 19].

Available data about the incidence of VAP recurrences in Covid-19 ARDS patients mentioned VAP recurrence rates between 30 and 46% [7, 10], which are far below the 81% recurrence rate we observed in our population. Interestingly the number of lung abscesses in our cohort (7% on first VAP, in total 19% of patients with VAP) was slightly higher compared to the incidence reported in other cohorts of Covid-19 patients with MV not restricted to ECMO [20–22], but not sufficient to explain our recurrence rate. Our higher rates of VAP and VAP recurrence could be explained by the particularity of our patients. First, by focusing on patients on ECMO support, we have selected patients with the most severe forms of ARDS, and unusually prolonged MV duration and ICU length of stay. Previous studies have shown that the risk of recurrence in this particular subset of patients is very high [5]. Secondly, ECMO circuit may alter disponibility of antibiotics, by decreasing their blood level [23, 24], and could therefore explain, at least partly, the high rate of persistent infections. However, this is highly unlikely, since we regularly monitored blood levels of antibiotics, and the proportion of patients with blood level below the EUCAST breakpoint of the pathogen responsible for VAP was low, and similar in patients with and without recurrence. Another hypothesis that may explain this high rate of recurrences is the pathophysiology of Covid-19 disease: endothelial dysfunction, endothelialitis and pulmonary vasculopathy are frequent [25]. Associated with dysregulated lung inflammation, this may enhance susceptibility to secondary bacterial infection and decrease antibiotic availability in the lung parenchyma, even when blood levels of antibiotics are in the targeted range [5].

Noticeably in the cohort by Gragueb-Chatti et al. which reported a 46% recurrence rate, 78% of first recurrences were caused by the same microorganism as the initial VAP, supporting our observation that most recurrences were relapses or persistent infections. However, a prolonged antimicrobial treatment duration did not seem to be associated with a lower VAP recurrence rate in our study. Although the absolute difference between the median treatment durations of each group was only 3 days, this data suggests that extending antimicrobial treatment duration might not be an adequate response to the high VAP recurrence risk in this population. Literature on VAP onset in Covid-19 ARDS suggest strategies for VAP prevention including digestive decontamination [26–28] and fighting lung microbiome disruption associated with Covid-19 [29], but studies focusing on optimizing VAP treatment or preventing relapse are lacking. Although not recommended by any recent guidelines, strategies such as antibiotic nebulization, combination therapy, or systematic bacteriological sampling at the end of the pre-planned duration of treatment to look for bacterial clearance, could be evaluated in the setting of Covid-19 patients on ECMO having developed VAP. However, one important message is that extending the duration of antimicrobial treatment for these patients doesn’t decrease the rate of recurrence, which reinforce the message on duration of antimicrobial treatment as a key strategy in antimicrobial stewardship programs [30]. Importantly, *Pseudomonas aeruginosa* as the pathogen responsible for VAP was an independent risk factor for VAP recurrence in our study. Since recent data show that short duration of treatment was not non-inferior to prolonged duration [31], and since controversies exist in the literature regarding duration of treatment for this pathogen [32, 33], issues regarding duration of treatment in this specific setting (namely Pseudomonas aeruginosa VAP in Covid-19 patients on ECMO) may be discussed. However, due to the small number of patients without Pseudomonas aeruginosa VAP recurrence in our cohort, we are unable to draw formal conclusion on this specific question, and a prolonged treatment may be discussed for these patients.

Our study has several limitations that should be underlined. Firstly, it is a monocentric retrospective study, therefore subjected to all potential bias associated with this kind of study, and this limits the impacts of the results. Then, its setting in an ECMO referral center, in conditions of limited resources when the epidemic peaked, resulted in a cohort of highly selected patients, not fully representative of the Covid-19–ARDS population. Secondly, data on antimicrobial consumption may be false, since we replaced missing data in patients who left the hospital before day 60 and were still alive at that time, assuming that these patients did not receive antibiotics. However, when replacing missing data using a worst-case scenario (assuming that these patients received antibiotics each day data were not available), we found similar results: median (IQR) antibiotic-free days were 11 (0–26) days in the short-duration group and one (0–19) days in the long-duration group (P = 0.03), thereby reinforcing our results.

## Conclusion

In patients with severe Covid-19–associated ARDS requiring ECMO support, VAP recurrences are frequent, driven by persistent infections and relapses, with Enterobacteriaceae and *Pseudomonas aeruginosa* as predominant causative microorganisms. Antimicrobial treatment ≥ 8 days of the first VAP episode did not seem to reduce the risk of VAP recurrence. Therapeutic strategies aiming at lowering VAP recurrence rate in these patients remain to be explored.

### Supplementary Information

**Additional file 1.** supplementary methods and results.

## Data Availability

The datasets generated during the current study are available from the corresponding author on reasonable request.

## Abbreviations

ARDS: Acute respiratory distress syndrome
BAL: Bronchoalveolar lavage
Covid-19: Coronavirus infection disease 2019
ECMO: Extracorporeal membrane oxygenation
ESBL: Extended-spectrum beta-lactamase
ICU: Intensive care unit
IQR: Interquartile range
MDRO: Multidrug-resistant organism
MV: Mechanical ventilation
SAPS: Simplified acute physiology score
SARS-CoV-2: Severe acute respiratory syndrome coronavirus 2
SOFA: Sequential organ-failure assessment
VAP: Ventilator-associated pneumonia
XDR: Extremely drug-resistant
