# Supplementary material for: Recurrent ventilator-associated pneumonia in severe Covid-19 ARDS patients requiring ECMO support
Source: Ann Intensive Care. 2024 Apr 25;14:67. doi: 10.1186/s13613-024-01295-1 (PMC11045714; doi:10.1186/s13613-024-01295-1)
Supplement: Supplementary file 1 — Additional file 1. supplementary methods and results. [file 13613_2024_1295_MOESM1_ESM.docx]

**Recurrent ventilator-associated pneumonia in severe Covid-19 ARDS patients requiring ECMO support**

Elena Collado-Lledó, Quentin Moyon, Juliette Chommeloux, Marc Pineton de Chambrun, Guillaume Hékimian, Ouriel Saura, David Lévy, Matthieu Schmidt, Alain Combes, Charles-Edouard Luyt, Lucie Le Fevre

**Online supplement**

**Supplemental Methods**

Serum dosage are routinely performed in our patients on ECMO for several antimicrobials, namely 3^rd^ generation cephalosporine, cefepime, fluoroquinolones, carbapenems, cotrimoxazole, anti MRSA agents (linezolide and vancomycin), ceftolozane/tazobactam, ceftazidim/avibactam. Dosages are not systematic and performed according to physicians’ prescription. Factors that may influence this prescription include duration of antimicrobial treatment (typically, determination was not performed if the planned end of treatment was before the result could be obtained), concomitant acute renal failure. All serum measurements are performed just before the next infusion and are hereafter referred as minimal concentration (Cmin). Anitimicrobial serum dosages for patients treated with narrow spectrum antimicrobial treatment as definitive treatment (i.e. amoxicillin/clavulanate, antistaphylococcal penicillin) are rarely, if never, performed.

Results of the dosages performed in our patients were retrospectively retrieved and analyzed. Patients were considered to have adequate serum level when this latter was above the European committee on antimicrobial susceptibility testing (EUCAST) breakpoints for the pathogen responsible for VAP. When VAP was due to multiple pathogens, the one with the highest breakpoint was used to considered whether or not Cmin was adequate.

**Supplemental Results**

Among the 210 patients with at least one VAP, 62 had an antibiotic serum level determination during the first VAP episode (eTable 2) , In 50 patients, antibiotic serum level were not considered because the patient received narrow spectrum antibiotic, or an antibiotic whose serum determination is not easily available (including amoxicillin/clavulanate, oxacillin, clindamycin, temocillin). Most patients treated with piperacillin, piperacillin/tazobactam and fluoroquinolones had no antibiotic serum level measurement performed because physicians considered that there are no need for routine measurement. Results of the antibiotic serum level determination are reported in eTable 2. Briefly, among the 45 patients with recurrence who had an antimicrobial serum determination, 41 (91%) were adequate, and among the 17 patients without recurrence who had an antimicrobial serum determination, 14 (82%) were considered adequate. One patient without recurrence had 2 determinations for the same antibiotic course: the first level was not adequate, the dosing was increased and a second determination performed 3 days later was adequate.

**eTable1**. Antimicrobial treatment of the 210 first ventilator-associated pneumonía according to recurrence or not*

|  | All patients  N = 210 | Recurrence  N = 171 | No recurrence  N = 39 | P value |
| --- | --- | --- | --- | --- |
| Appropriate empiric treatment | 194 (92) | 158 (92) | 36 (92) | 0.9 |
| Empirical treatment |  |  |  |  |
| Main agent  Aminopenicillin + clavulanate  Antistaphylococcal penicillin  3rd generation cephalosporin  Antipseudomonal cephalosporin †  Fluroroquinolone  Anti MRSA ‡  Piperacillin/tazobactam  Carbapenem | 4 (2)  6 (3)  25 (12)  62 (30)  7 (3)  5 (2)  87 (41)  14 (7) | 4 (2)  4 (2)  22 (13)  52 (30)  6 (4)  5 (3)  63 (40)  9 (5) | 0  2 (5)  3 (8)  10 (26)  1 (3)  0  18 (46)  5 (13) | 0.4 |
| Combination with an aminoglycosides | 67 (32) | 52 (30) | 15 (38) | 0.3 |
| Anti MRSA ‡, alone or with a betalactam | 21 (10) | 15 (9) | 6 (15) | 0.2 |
| Definitive treatment |  |  |  |  |
| Molecule  Aminopenicillin + clavulanate  Antistaphylococcal penicillin  3rd generation cephalosporin  Antipseudomonal cephalosporin †  Temocillin  Ceftolozane/tazobactam  Ceftazidime/avibactam  Fluroroquinolone  Anti MRSA ‡  Piperacillin  Piperacillin/tazobactam  Carbapenem  Aztreonam  Cotrimoxazole  Clindamycin  Aztreonam + avibactam  Fluoroquinolone + cotrimoxazole | 26 (12)  21 (10)  28 (13)  26 (12)  2 (1)  1 (0.5)  1 (0.5)  19 (9)  7 (3)  41 (20)  11 (5)  20 (10)  1 (0.5)  2 (1)  1 (0.5)  2 (1)  1 (0.5) | 21 (12)  19 (11)  23 (13)  19 (11)  2 (1)  1 (1)  0  16 (9)  6 (4)  36 (21)  8 (5)  14 (8)  1 (1)  1 (1)  1 (1)  2 (1)  1 (1) | 5 (13)  2 (5)  5 (13)  7 (18)  0  0  1 (3)  3 (8)  1 (3)  5 (13)  3 (8)  6 (15)  0  1 (3)  0  0  0 | 0.6 |
| Combination | 4 (2) | 4 (2) | 0 | 0.3 |
| Betalactam + anti MRSA agent | 6 (3) | 5 (3) | 1 (3) | 0.9 |

* excluding patients with lung abscess

† ceftazidime or cefepime

† vancomycin or linezolide

**eTable 2**. Proportion of patients with adequate* antibiotic serum level

| Definitive treatment | All patients  N = 210 | Patients with recurrence  N = 171 | Patients without recurrence  N = 39 |
| --- | --- | --- | --- |
| Molecule received  Aminopenicillin + clavulanate  Dosage performed  Dosage considered adequate  Antistaphylococcal penicillin  Dosage performed  Dosage considered adequate  3rd generation cephalosporin  Dosage performed  Dosage considered adequate  Ceftazidime  Dosage performed  Dosage considered adequate  Cefepime  Dosage performed  Dosage considered adequate  Temocillin  Dosage performed  Dosage considered adequate  Ceftolozane/tazobactam  Dosage performed  Dosage considered adequate  Ceftazidime/avibactam  Dosage performed  Dosage considered adequate  Fluroroquinolone  Dosage performed  Dosage considered adequate  Anti MRSA agent‡  Dosage performed  Dosage considered adequate  Piperacillin  Dosage performed  Dosage considered adequate  Piperacillin/tazobactam  Dosage performed  Dosage considered adequate  Carbapenem  Dosage performed  Dosage considered adequate  Aztreonam  Dosage performed  Dosage considered adequate  Cotrimoxazole  Dosage performed  Dosage considered adequate  Clindamycin  Dosage performed  Dosage considered adequate  Aztreonam + avibactam  Dosage performed  Dosage considered adequate | 26  0  -  21  0  -  28  7  5  6  4  2  20  16  15  2  0  -  1  1  1  1  1  1  19  4  4  7  7  7  41  2  1  11  0  -  20  14  14  1  0  -  3  3  1  1  0  -  2  2  2 | 21  0  -  19  0  -  23  5  4  4  2  2  15  12  11  2  0  -  1  1  1  0  -  -  16  3  3  6  6  6  36  1  1  8  0  -  14  10  11  1  0  -  2  2  1  1  0  -  2  2  2 | 5  0  -  2  0  -  5  2  1  2  2  2  5  4  4  0  -  -  0  -  -  1  1  1  3  1  1  1  1  1  5  1†  1†  3  0  -  6  4  3  0  -  -  1  1  0  0  -  -  0  -  - |

- Antibiotic serum level was considered adequate when it was above the European committee on antimicrobial susceptibility testing (EUCAST) breakpoints for the corresponding pathogen.

† One patient had 2 piperacillin serum measurements for the same VAP episode. The first measurement was below the therapeutic range; dosage was increased and the second measurement was above the therapeutic level.

eFigure 1:
